# Ypd1 Is an Essential Protein of the Major Fungal Pathogen *Aspergillus fumigatus* and a Key Element in the Phosphorelay That Is Targeted by the Antifungal Drug Fludioxonil

**DOI:** 10.3389/ffunb.2021.756990

**Published:** 2021-10-18

**Authors:** Sebastian Schruefer, Anja Spadinger, Christoph Kleinemeier, Laura Schmid, Frank Ebel

**Affiliations:** Department of Veterinary Sciences, Institute for Infectious Diseases and Zoonoses, Chair for Bacteriology and Mycology, Ludwig-Maximilians-University, Munich, Germany

**Keywords:** Ypd1, TcsC, HOG pathway, HPt protein, fludioxonil, Skn7

## Abstract

*Aspergillus fumigatus* is a major fungal pathogen causing life threatening infections in immunocompromised humans and certain animals. The HOG pathway is for two reasons interesting in this context: firstly, it is a stress signaling pathway that contributes to the ability of this pathogen to adapt to various stress conditions and secondly, it is the target of antifungal agents, such as fludioxonil or pyrrolnitrin. In this study, we demonstrate that Ypd1 is an essential protein in *A. fumigatus*. As the central component of the multistep phosphorelay it represents the functional link between the sensor histidine kinases and the downstream response regulators SskA and Skn7. A GFP-Ypd1 fusion was found to reside in both, the cytoplasm and the nucleus and this pattern was only slightly affected by fludioxonil. A strain in which the *ypd*1 gene is expressed from a tet-on promoter construct is unable to grow under non-inducing conditions and shows the characteristic features of *A. fumigatus* wild type hyphae treated with fludioxonil. Expression of wild type Ypd1 prevents this lethal phenotype, but expression of an Ypd1 mutant protein lacking the conserved histidine at position 89 was unable to do so, which confirms that *A. fumigatus* Ypd1 is a phosphotransfer protein. Generation of *ypd*1^tet−on^ variants of several mutant strains revealed that the lethal phenotype associated with low amounts of Ypd1 depends on SskA, but not on TcsC or Skn7. The Δ*ssk*A *ypd*1^tet−on^, but not the Δ*ssk*AΔ*skn*7 *ypd*1^tet−on^ mutant, was sensitive to fludioxonil, which underlines the importance of Skn7 in this context. We finally succeeded to delete *ypd*1, but only if *ssk*A and *skn*7 were both inactivated, not in a Δ*ssk*A single mutant. Hence, a deletion of *ypd*1 and an inactivation of Ypd1 by fludioxonil result in similar phenotypes and the two response regulators SskA and Skn7 are involved in both processes albeit with a different relative importance.

## Introduction

The High Osmolarity Glycerol (HOG) pathway is a major fungal signaling cascade. Initially found to control the response to osmotic stress (Brewster et al., 1993), the HOG pathway was later on shown to process a variety of stress signals and to contribute to the virulence of several fungal pathogens (Ma and Li, 2013; Román et al., 2020).

Most information on the HOG pathway originates from work done with *Saccharomyces cerevisiae* (Hohmann, 2002). In the absence of osmotic stress, the membrane-bound sensing kinase Sln1p phosphorylates the histidine phosphotransfer (HPt) protein Ypd1p that transfers phosphoryl groups to the response regulator Ssk1p. If a stress signal is perceived, Sln1p becomes inactive and this results in a dephosphorylation of Ssk1p and, in turn, in an activation of Ssk2p and the HOG MAP kinase module (Posas and Saito, 1998; Horie et al., 2008; Fassler and West, 2013). This organization explains the lethal consequences of a *SLN*1 or *YPD*1 deletion, since a permanent inactivation or the absence of either protein entails a persistent and lethal activation of Hog1p (Maeda et al., 1994). This concept is corroborated by the finding that *YPD*1 deletions in *S. cerevisiae* or *Cryptococcus neoformans* are not lethal in strains, in which the HOG pathway is inactivated by an additional mutation (Posas et al., 1996; Lee et al., 2011). The fact that some Ypd1p orthologs are not essential (Nguyen et al., 2000; Lee et al., 2011; Mavrianos et al., 2014; Jacob et al., 2015; Rodríguez-González et al., 2017) points to differences in the Ypd1-dependent signaling processes in different fungi.

Ypd1 is part of a multistep phosphorelay that also comprises one or more hybrid histidine kinases (HHKs) and two response regulators (RRs) (Defosse et al., 2015) (compare **Figure 7**). The perceived signal is relayed by a series of phosphorylation events: starting with an autophosphorylation of a His residue in the His Kinase acceptor (HisKA) domain, the phosphoryl group is then transferred intramolecularly to an Asp residue in the receiver domain of the HHK. In the next step, the phosphoryl group is transferred to a conserved His residue in the HPt protein Ypd1 and Ypd1 finally phosphorylates an Asp residue in the receiver domain of the RRs. This relay was first described for *S. cerevisiae* (Maeda et al., 1994; Posas et al., 1996) and represents the blueprint for the phosphorelays of other fungi.

HHKs represent a fungal-specific form of two-component systems (TCS) that represent sensor molecules that allow microorganisms to sense and respond to a plethora of environmental signals and stress conditions. Bacterial TCS consist of a membrane-bound histidine kinase and an intracellular response regulator protein, whereas fungal HHKs integrate both TCS functions in one protein (Bahn, 2008).

Most filamentous fungi harbor several HHKs and Catlett et al. (2003) defined 11 different groups. All HHKs share a common C-terminal signaling module, but each group is characterized by a distinct N-terminal sensing module that is specialized in the measurement of certain environmental signals. Remarkably, all fungi analyzed so far harbor one HPt protein and two RRs and the paradigms of these proteins were identified and characterized in *S. cerevisiae* (Brown et al., 1994; Li et al., 1998).

*Aspergillus fumigatus* is a major air-borne fungal pathogen that causes severe and often life-threatening infections in immunocompromised patients (McCormick et al., 2010) and patients suffering from severe viral infections, such as those caused by influenza or SARS-CoV-2 (Dewi et al., 2021). Although it lacks sophisticated virulence traits, *A. fumigatus* is a successful pathogen and this is largely due to its stress resistance and ability to rapidly adapt to a wide range of environmental conditions (Tekaia and Latgé, 2005; McCormick et al., 2010). This versatility is to a large extend attributable to a set of 13 HHKs that belong to eight different groups of HHKs (Defosse et al., 2015). One of them, the group III HHK TcsC, controls the activity of the HOG pathway (McCormick et al., 2012; Hagiwara et al., 2013) and thereby resembles Sln1p at the functional level. A remarkable difference exists with regards to the viability of respective deletion mutants: The group VI HHK Sln1p of *S. cerevisiae* is essential (Maeda et al., 1994), whereas TcsC and group III HHKs of other filamentous fungi are not (Defosse et al., 2015). The pivotal role of Sln1p reflects the fact that it is the sole HHK in baker's yeast and essentially required to prevent an uncontrolled and lethal activation of the HOG pathway. The existence of multiple HHKs in most other fungi seems to provide a level of functional redundancy that enables group III HHK mutants to thrive under standard growth conditions.

The sensing module of group III HHKs consists of several HAMP domains that control the activity of the C-terminal signaling or kinase module, which after autophosphorylation may transfer a phosphoryl group to the HPt protein Ypd1. Expression of a truncated form of TcsC lacking its sensing module causes a dysregulation of the osmotic homeostasis in *A. fumigatus* and results in a fatal influx of water (Spadinger and Ebel, 2017), which emphasizes the necessity for a tight control of the activity of the TcsC kinase module.

In *A. nidulans*, a model organism for filamentous fungi, the HOG pathway controls the response to several stress conditions (Vargas-Pérez et al., 2007; Hagiwara et al., 2009). Mutants in genes encoding the group III HHK NikA, which is assumed to phosphorylates YpdA, are viable (Hagiwara et al., 2007; Vargas-Pérez et al., 2007), but attempts to generate an *ypd*A deletion mutant failed, suggesting that YpdA is an essential protein in *A. nidulans* (Furukawa et al., 2005; Vargas-Pérez et al., 2007; Yoshimi et al., 2021) demonstrated very recently that downregulation of *ypd*A expression results in growth inhibition and aberrant hyphal morphology. Although related, *A. nidulans* and *A. fumigatus* show differences in the phenotypes of their respective group III HHK mutants (Böhmer et al., 2020) that may derive from differences in the biological activities of their HPt proteins.

To characterize the phosphorelay in *A. fumigatus*, we have analyzed its individual components using deletion mutants, point mutations and GFP fusion proteins. Our data demonstrate that Ypd1 is an essential protein in *A. fumigatus* and that antifungal agents, such as fludioxonil, kill *A. fumigatus* by a TcsC-mediated inactivation of Ypd1. Further evidence suggests that the activity of *A. fumigatus* Ypd1 is controlled by at least two different HHKs.

## Materials and Methods

### Strains

Strain AfS35 is a nonhomologous end-joining-deficient derivative of the clinical *A. fumigatus* isolate D141 (Krappmann et al., 2006). All mutant strains used in this study are summarized in the Supplementary Table 1 and were routinely grown in Aspergillus Minimal Medium (AMM).

### Generation of Mutant Strains

The oligonucleotides used in this study are summarized in the Supplementary Table 2. All fragments that were subsequently used in cloning experiments were amplified with the Q5 High Fidelity polymerase (New England Biolabs). All constructs were verified by sequencing and all strain were verified by analytic PCR reactions.

To generate a GFP-Ypd1 fusion construct, the *ypd*1 sequence was amplified from chromosomal DNA of strain AfS35 using oligonucleotides GFP-ypd1-For and ypd1-Rev. The resulting fragment was cloned into the EcoRV site of pSK379-gfp (harboring a pyrithiamine resistance cassette) and subsequently transformed into AfS35. Details on the construction of pSK379 and pSK379-gfp have been published previously (Wagener et al., 2008; Szewczyk and Krappmann, 2020). Fusion proteins of *ssk*A and *skn*7 were generated by cloning the respective PCR products generated with oligonucleotide pairs sskA-For/sskA-Rev and GFP-skn7-For/GFP-skn7-Rev into the PmeI and EcoRV site of pSK379, respectively. The resulting plasmids encoding *ssk*A-*gfp* and *gfp*-*skn*7 were then transformed into protoplasts of strain AfS35.

To allow controlled expression, we replaced the native *ypd*1 promoter by a tet-on promoter construct. We amplified a 892 bp fragment upstream of the *ypd*1 gene using oligonucleotides Ypd1-up-For and Ypd1-up-Rev-tet-on. A second 922 bp fragment, starting with the ATG of the *ypd*1 gene and comprising additionally 290 bp downstream of *ypd*1 was amplified with oligonucleotides ypd1-For-SfiI and ypd1-do-Rev-tet-on. Using oligonucleotide-derived SfiI sites, both fragments were ligated to the 4.3 kb tet-on cassette obtained from plasmid pYZ002 by SfiI digestion (Helmschrott et al., 2013; Neubauer et al., 2015). The resulting construct was then introduced into AfS35 and several AfS35-derived deletion mutants. The resulting strains were routinely propagated in medium containing 5 μg/ml doxycyclin.

To visualize nuclei, we amplified a fragment of 468 bp corresponding to the C-terminal end of the StuA protein of *A. fumigatus* (Afu2g07900) using oligonucleotides stuA-in-EcoRV-FOR and stuA-in-EcoRV-REV. The amplicon was subsequently cloned into the EcoRV site of pSK379-rfp in which the original pyrithiamine resistance cassette had been replaced by a hygromycin cassette derived from pSILENT.

An *ypd*1 deletion construct was generated using a fusion PCR approach. The up- und downstream regions flanking the *ypd*1 gene were amplified using oligonucleotides ypd1-deletion-up-For/ypd1- deletion-up-REV, ypd1-deletion-do-For/ypd1-deletion-do-Rev and Pyrith-cassette-For/Pyrith-cassette-Rev. The construct was introduced in protoplasts of the target strain and pyrithiamine resistant clones were further characterized.

To introduce point mutations into Ypd1, we have first cloned the *ypd*1 gene, which was amplified with oligonucleotides ypd1-For and ypd1-Rev from chromosomal DNA of AfS35, into the PmeI site of pSK379. In the next step, we used the Q5 Site-Directed Mutagenesis Kit (New England Biolabs) and oligonucleotides designed using the NEBaseChanger software (https://nebasechanger.neb.com/) to generate the desired point mutation. The Ypd1 H89G construct was obtained using oligonucleotide pair ypd1-H89G-For/ypd1-H89G-Rev. The constructs harboring the wild type or the mutated gene were verified by sequencing and the plasmids were then introduced into protoplasts of AfS35 *ypd*1^tet−on^.

Alignments of protein sequence were performed with MUSCLE (https://www.ebi.ac.uk/Tools/msa/muscle/). DNA sequences were aligned using Clustal Omega (https://www.ebi.ac.uk/Tools/msa/clustalo/). Protein sequence were analyzed for domains and motifs using SMART (http://smart.embl-heidelberg.de/).

### qPCR

For quantification of *ypd*1 expression, RNA was isolated from 3 ml cultures in AMM containing 0.5 μg/ml doxycyclin that were inoculated with 1.5 × 10^6^ conidia and subsequently incubated at 37°C for 12 h. RNA was isolated using the InnuPrep Plant RNA Kit (Analytik Jena, Jena, Germany) combined with on-column DNase I digestion (Analytik Jena) according to instructions of the vendor. cDNA was synthesized from these samples using the High-Capacity cDNA Reverse Transcriptation Kit (ThermoFisher, Waltham, MA, USA) and qPCR runs were performed with 5XEvaGreen®Mastermix (Bio&Sell, Feucht, Germany) using a QuantStudio5 qPCR system (ThermoFisher). After an initial denaturation for 15 min at 95°C, 40 PCR cycles followed (denaturation for 15 s at 95°C, annealing for 20 s 60°C and elongation for 20 s at 72°C). Expression levels of *ypd*1 were normalized to the expression of the *tub*A gene and the expression level of the control strain AfS35 *ypd*1^tet−on^ according to the ΔΔCt method.

### Phenotypic Characterization

For drop dilution assays, freshly isolated conidia were counted using a Neubauer improved chamber and series of ten-fold dilutions starting with 2 × 10^7^ conidia per ml were spotted onto AMM plates in aliquots of 2.5 μl. If not stated otherwise, plates supplemented with the indicated agents were incubated for 48 h at 37°C and sorbitol-containing plates were incubated for 72 h.

### Microscopy

To determine the spatial distribution of GFP fusion proteins, we grew the respective strains in AMM in cell culture multi-well chambers (IBIDI, Martinsried, Germany). Vital hyphae were analyzed using a confocal laser scanning microscope LSM880 (Zeiss). For bleaching experiments a part of the sample (indicated as a boxed area in the respective image) was bleached with 488 nm laser light until the GFP signal was quantitatively gone. GFP fluorescence was quantified using the Zeiss ZEN Blue software.

For microscopic images, hyphae were grown on glass cover slips in AMM with and without doxy and subsequently fixed with 3.7% formaldehyde/PBS for 5 min. After staining with CFW (3.5 μg/ml in PBS) for 5 min, samples were washed and mounted using VectaShield mounting medium. Images from colonies grown on agar plates were taken using a Leica DM5000B microscope with an attached DFC3000G camera. For further microscopic characterization, colonies grown on AMM plates were cut-out and the resulting agar blocks were further trimmed to obtain a thin slice with the colony on its surface. The sample was transferred in a 12 well plate and incubated for 10 min with 2 ml CFW solution (3.5 μg/ml in PBS). After two washing steps in 2 ml PBS for 3 min, the samples were placed on glass specimen slide, covered with a glass cover slip and inspected using a Leica DM5000B microscope equipped with a DFC 3000G camera.

To detect lysis of hyphal cells, the AfS35 ypd1^tet−on^ strain was grown on glass cover slips in AMM. At the indicated time point, the volume of medium per well of a 24 well plate was reduced to 500 and 5 μl of a 10 mM stock solution of CellTracker™ Blue CMAC (7-amino-4-chloromethylcoumarin) (ThermoFisher) in DMSO were added. The plate was then incubated for 30 min at RT, the cover slips were washed once with 1 ml PBS and then directly analyzed using a Leica DM5000B microscope.

## Results

### Ypd1 Shuttles Between the Cytoplasm and the Nucleus of *A. fumigatus*

**Figure 1:**
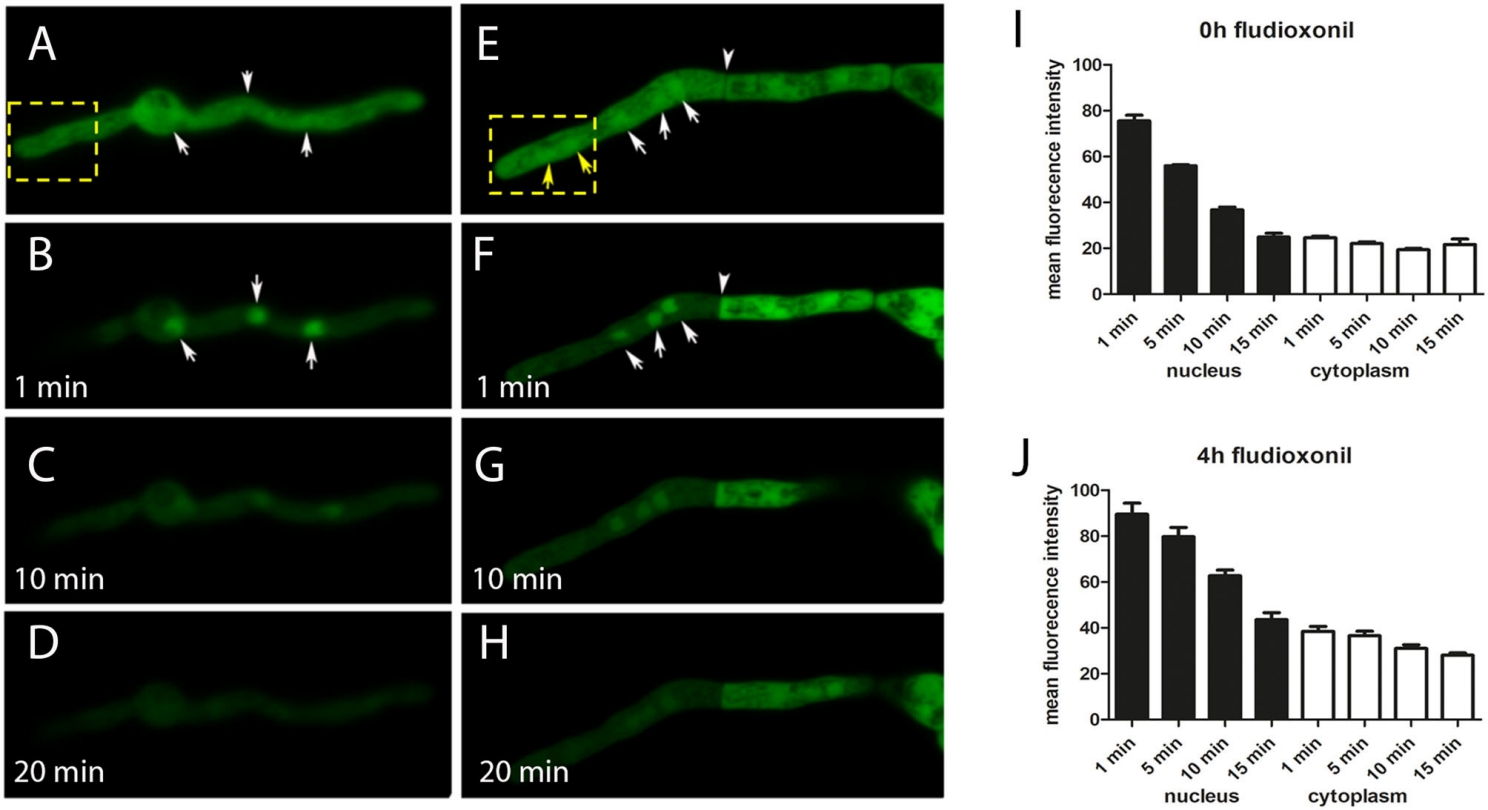
Localization of GFP-Ypd1 in *A. fumigatus*. The spatial distribution of the fusion protein in normal hyphae of strain AfS35 and 4 h after addition of 1 μg/ml fludioxonil is shown in **(A)** and **(E)**, respectively. The depicted hyphae were then bleached (the corresponding areas are indicated by yellow boxes). This treatment let to a disappearance of the cytosolic GFP signals in the whole hypha or compartment, depending on the condition of the septal pore. Nuclei in this zone also lost their GFP fluorescence (**F**, yellow arrows), but the GFP-signals in those nuclei that were not exposed to the bleaching were not affected (**A,B,E,F**, white arrows). The relative enrichment of the GFP-signals in these nuclei disappeared over time **(C**/**D** and **G**/**H)**. The arrowhead in **(F)** points to a septum that was closed in response to fludioxonil. **(A–H)** Show single optical planes of confocal images. **(I,J)** Show a quantification of GFP fluorescence in nuclei (closed bars) and cytoplasm (open bars) of control hyphae **(I)** and hyphae treated with fludioxonil for 4 h **(J)**.

In order to analyze the localization of Ypd1, we expressed a GFP-Ypd1 fusion protein in *A. fumigatus* strain AfS35. Under ambient conditions, this protein showed a homogenous nucleo-cytoplasmic distribution (Figure 1A). Bleaching of the cytosolic GFP-Ypd1 pool resulted in a strong enrichment of the GFP signals in the nuclei (Figure 1B). This distinct pattern became weaker over time and was hardly detectable after 10 min (Figures 1C,D) indicating that GFP-Ypd1 shuttles between the nucleus and the cytoplasm. After fludioxonil treatment, the spatial distribution of GFP-Ypd1 was not substantially different, although we observed a slightly stronger nuclear enrichment of the GFP fluorescence after 4 h treatment (Figure 1E). This is particular evident in Supplementary Figure 1 that depicts hyphae that were additionally stained with the DNA-specific dye DAPI. However, this nuclear enrichment was not always as clearly detectable. Bleaching of the cytosolic fusion proteins in fludioxonil-treated hyphae revealed a similar dynamics of GFP-Ypd1 as in untreated hyphae (Figures 1F–H). Quantification of the nuclear and cytosolic GFP fluorescence corroborated this finding (Figures 1I,J). The two RRs SskA and Skn7 showed distinct and complementary localization patterns: SskA was only detectable in the cytoplasm, whereas Skn7 was exclusively found in the nuclei. Both patterns were not affected by fludioxonil (Supplementary Figure 2). In conclusion, our data show a similar spatial distribution of Ypd1, SskA and Skn7 as it was previously reported for their orthologous proteins in *S. cerevisiae* (Lu et al., 2003).

### Reduced Expression of *Ypd1* Causes a Severe Growth Defect in *A. fumigatus*

**Figure 2:**
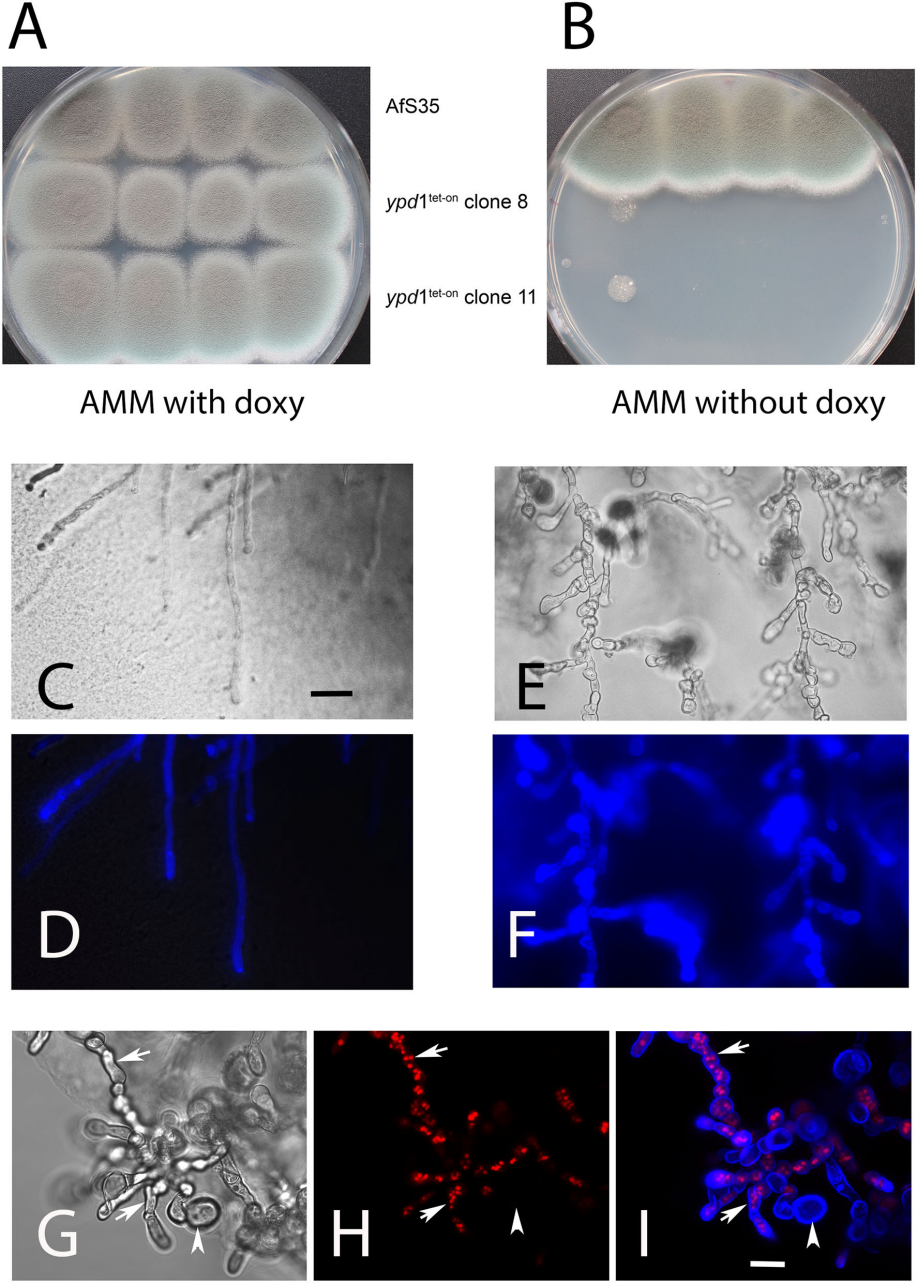
Depletion of Ypd1 is lethal for *A. fumigatus*. We have analyzed the growth of two clones, in which the *ypd*1 gene was cloned under the control of a tet-on promoter construct (AfS35 *ypd*1^tet−on^) and the parental strain AfS35 on AMM plates with and without doxycyclin (**A**,**B**, respectively). Under non-inducing conditions, the growth of the mutants is strongly impaired **(B)**. Microscopic images of hyphae from the edges of these colonies are shown in **(C**–**I)**. The hyphae of the parental strain show a normal morphology and a normal CFW staining **(C,D)**. The mutant hyphae are short, swollen and contain many septa **(E,G)**. A stronger chitin staining of mutant hyphae with CFW is evident from **(F)** and **(I)** (compared to the control staining in **D**). The swollen compartments of mutant hyphae harbor elevated numbers of nuclei [arrows in **(H,I)**]. Some of these compartments were already lysed and lacked nuclei [arrowhead in **(H,I)**]. The bars shown in **(C**,**F)** represent 30 and 10 μm, respectively, and are valid for **(C–F)** and **(G–I)**. The images shown in **(D,F,H,I)** are maximum projects of z-stacks.

The HPt proteins of *S. cerevisiae* and *A. nidulans* were previously reported to be essential (Posas et al., 1996; Vargas-Pérez et al., 2007). We therefore started our analysis by replacing the native promoter of the *ypd*1 gene in *A. fumigatus* strain AfS35 by a tet-on controlled promoter construct. Two independent AfS35 *ypd*1^tet−on^ clones (#8 and #11) were further characterized. Both mutants grew well in the presence of doxycyclin (doxy) (Figure 2A), but showed a severe growth defect under non-inducing conditions (Figure 2B). Microscopic inspection of the resulting compact mutant colonies obtained after 48 h revealed short and swollen hyphae that were strongly stained by the chitin-specific dye CFW and contained many septa (Figures 2E,F). In contrast, hyphae from colonies grown in the presence of doxy had a normal morphology, a normal septation pattern and a moderate CFW staining (Figures 2C,D). We also expressed RFP-StuA in AfS35 *ypd*1^tet−on^ to visualize the nuclei. Using this strain, we found that the swollen compartments induced by Ypd1 depletion contained either high numbers of nuclei or were devoid of RFP fluorescence (Figures 2G–I, arrows and arrowhead, respectively). In conclusion, we found that a reduced expression of *ypd*1 results in a lethal phenotype, similar to that described for fludioxonil-treated *A. fumigatus* wild type hyphae (McCormick et al., 2012; Hagiwara et al., 2013).

**Figure 3:**
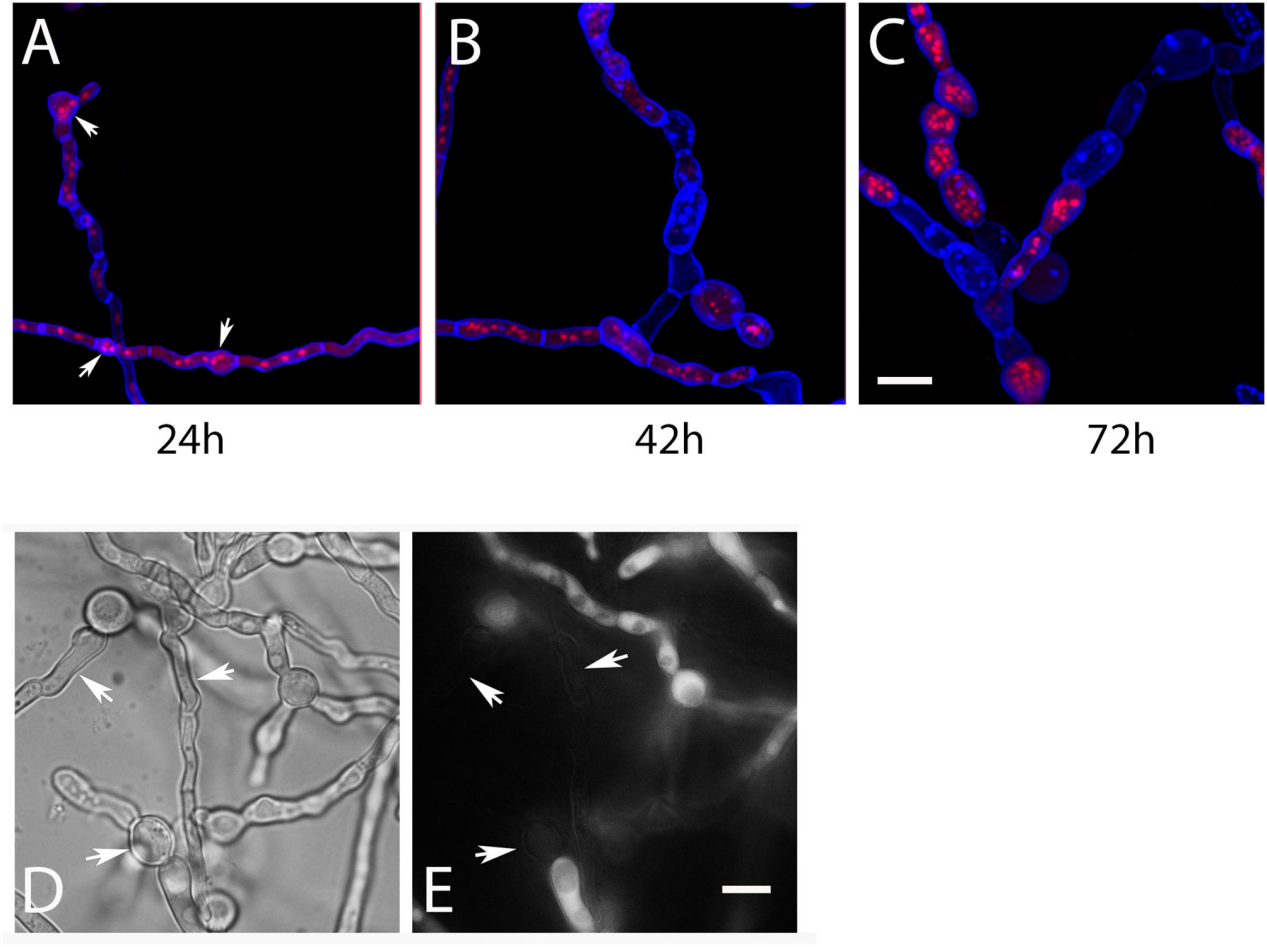
Hyphae of AfS35 *ypd*1^tet−on^ expressing RFP-StuA to visualize the nuclei were grown in AMM liquid medium without doxy and images were taken at the indicated time points. Accumulations of nuclei were detectable after 24 h in some compartments (arrows in **A)**. After longer incubation, the swelling of the fungal cells became more pronounced and lysis of certain compartments is evident by a loss of RFP-StuA-positive nuclei **(B,C)**. Images are maximum projections of z-stacks taken with a confocal microscope. **(D,E)** Show the AfS35 *ypd*1^tet−on^ strain after 72 h growth in liquid AMM without doxy. The CMAC-stained cytoplasm is shown in **(E)**. Arrows indicate compartments that are devoid of cytoplasm. The bars in **(C)** and **(E)** represent 10 μm and are valid for **(A**–**C)** and **(D,E)**, respectively.

The microscopic data described so far were obtained with hyphae grown on agar plates, but we also analyzed RFP-StuA-expressing AfS35 *ypd*1^tet−on^ hyphae that were grown in liquid AMM. After 24 h at 37°C without doxy these hyphae showed a normal morphology and CFW staining, but small accumulations of nuclei were already detectable in some cells (Figure 3A, arrows). After 42 and 72 h in medium without doxy, the hyphae showed an aberrant and swollen morphology, contained elevated numbers of nuclei and were strongly stained with CFW (Figures 3B,C). We used the dye CMAC to stain the cytoplasm of AfS35 *ypd*1^tet−on^ hyphae after 72 h in liquid AMM without doxy; intact compartments were labeled, whereas other compartments showed no CMAC staining indicating a loss of cytoplasm (Figures 3D,E). This indicates that a reduced level of Ypd1 finally results in hyphal lysis. The conidia that were used in these experiments were harvested from plates containing doxy. We assume that the Ypd1 proteins that were present in these spores enabled the limited growth. At later time points, the cellular Ypd1 content apparently declined below a critical value, which resulted in morphological changes that are also characteristic for fludioxonil-treated *A. fumigatus* wild type cells.

A titration of the doxy concentration revealed that the AfS35 *ypd*1^tet−on^ strain grew well at concentrations above 1 μg/ml, whereas concentrations of 0.01–0.5 μg/ml allowed only a restricted growth. In the presence of fludioxonil, the growth of all colonies was severely impaired and reduced to a level also found under non-inducing conditions (Supplementary Figure 3). This indicates that any potential growth due to the presence of doxy was prevented by the antifungal activity of fludioxonil. This finding provides further support for the concept that fludioxonil kills hyphae by an inactivation of Ypd1.

### The Conserved His Residue at Position 89 Is Essential for the Biological Activity of Ypd1

**Figure 4:**
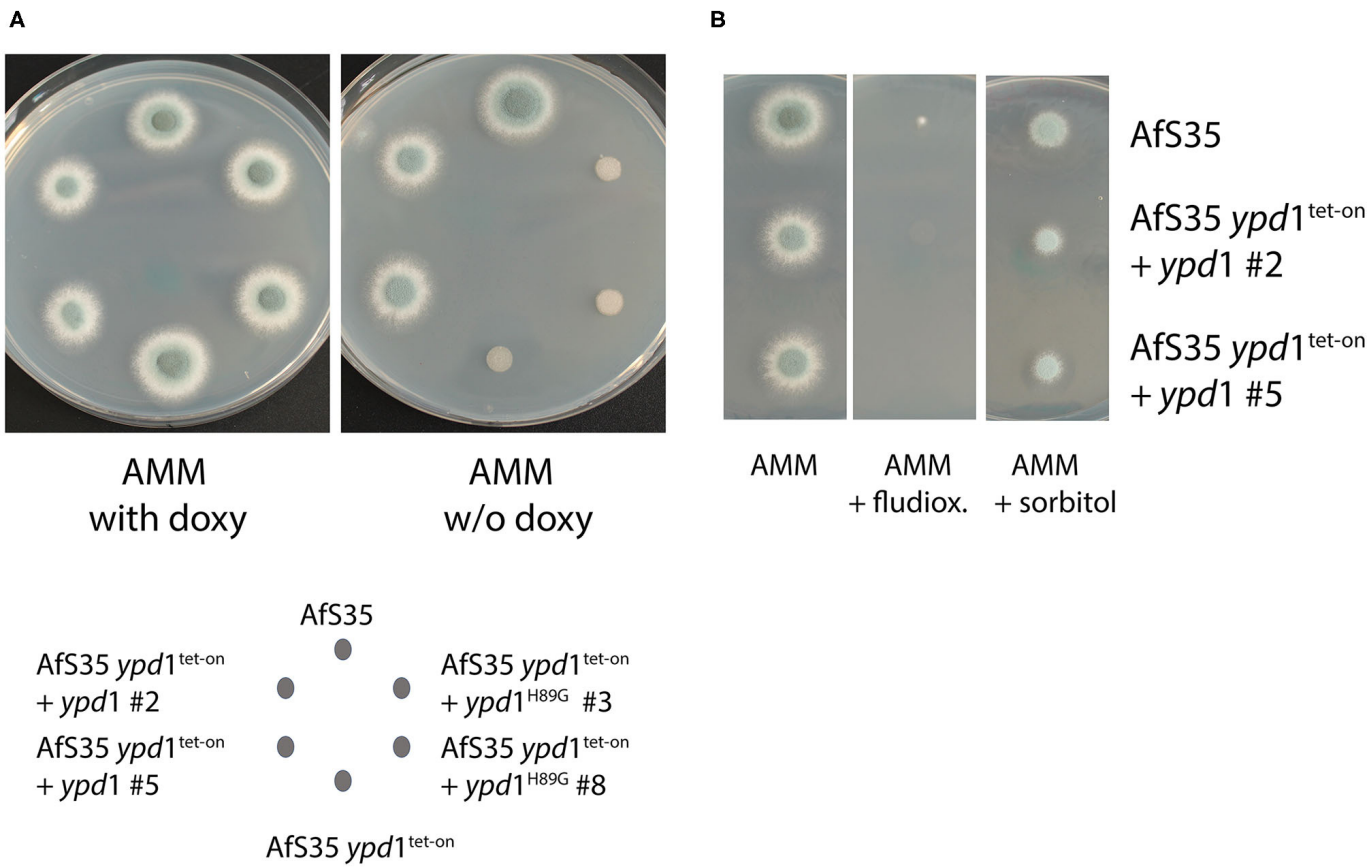
The conserved His residue of the HPt domain is essential for the biological function of *A. fumigatus* Ypd1. We expressed the native *ypd*1 gene or its derivative encoding Ypd1^H89G^ in the AfS35 *ypd*1^tet−on^ mutant. Two clones of each mutant strain were tested. On AMM plates without doxy, the *ypd*1 wild type gene, but not the mutated form restored the growth of the AfS35 *ypd*1^tet−on^ strain **(A)**. The wild type allele furthermore let to a sensitivity to fludioxonil (1 μg/ml) and enabled growth of the mutant in the presence of 1.2 M sorbitol **(B)**. Each colony was derived from a spot containing 5 × 10^4^ conidia applied in 2.5 μl.

The conserved histidine residues at position 64 of *S. cerevisiae* Ypd1p and position 85 of *A. nidulans* YpdA were previously identified as essential phosphorylation sites of the HisKA domain of these HPt proteins (Posas et al., 1996; Azuma et al., 2007). We therefore mutagenized the triplet encoding the corresponding His_89_ residue of *A. fumigatus* Ypd1 (compare Supplementary Figure 4). Expression of the resulting Ypd1^H89G^ mutant and the Ypd1 wild type protein in AfS35 *ypd*1^tet−on^ enabled us to compare the biological activity of both proteins. In the presence of doxy, both strains grew well (Figure 4A), but only the wild type protein was able to overcome the growth defect of the parental strain AfS35 *ypd*1^tet−on^ on plates without doxy (Figure 4A). To rule out that these results reflect differences in gene expression, we performed a qPCR analysis for *ypd*1 in AfS35 *ypd*1^tet−on^ and the two complemented strains. Since we obtained only RNA of poor quality from hyphae grown in the absence of doxy, we performed the experiments in the presence of 0.5 μg/ml doxy, which allowed the formation of sporulating colonies (compare Supplementary Figure 3). Both complemented strains showed a higher expression of *ypd*1 than the parental strain AfS35 *ypd*1^tet−on^. The expression rates of the two complemented strains were similar indicating that the observed phenotypic differences are not the results of different expression levels of *ypd*1 (Supplementary Figure 5). Thus, expression of wild type Ypd1 restored growth and the resulting hyphae were sensitive to fludioxonil and able to grow like wild type in the presence of 1.2M sorbitol, whereas the mutated Ypd1^H89G^ protein was, in contrast, unable to restore the fungal growth (Figure 4B). In conclusion, these data demonstrate that His_89_ is essentially required for the biological activity of *A. fumigatus* Ypd1.

### SskA Is Essential for the Deleterious Effect Resulting From a Reduced *ypd1* Expression

**Figure 5:**
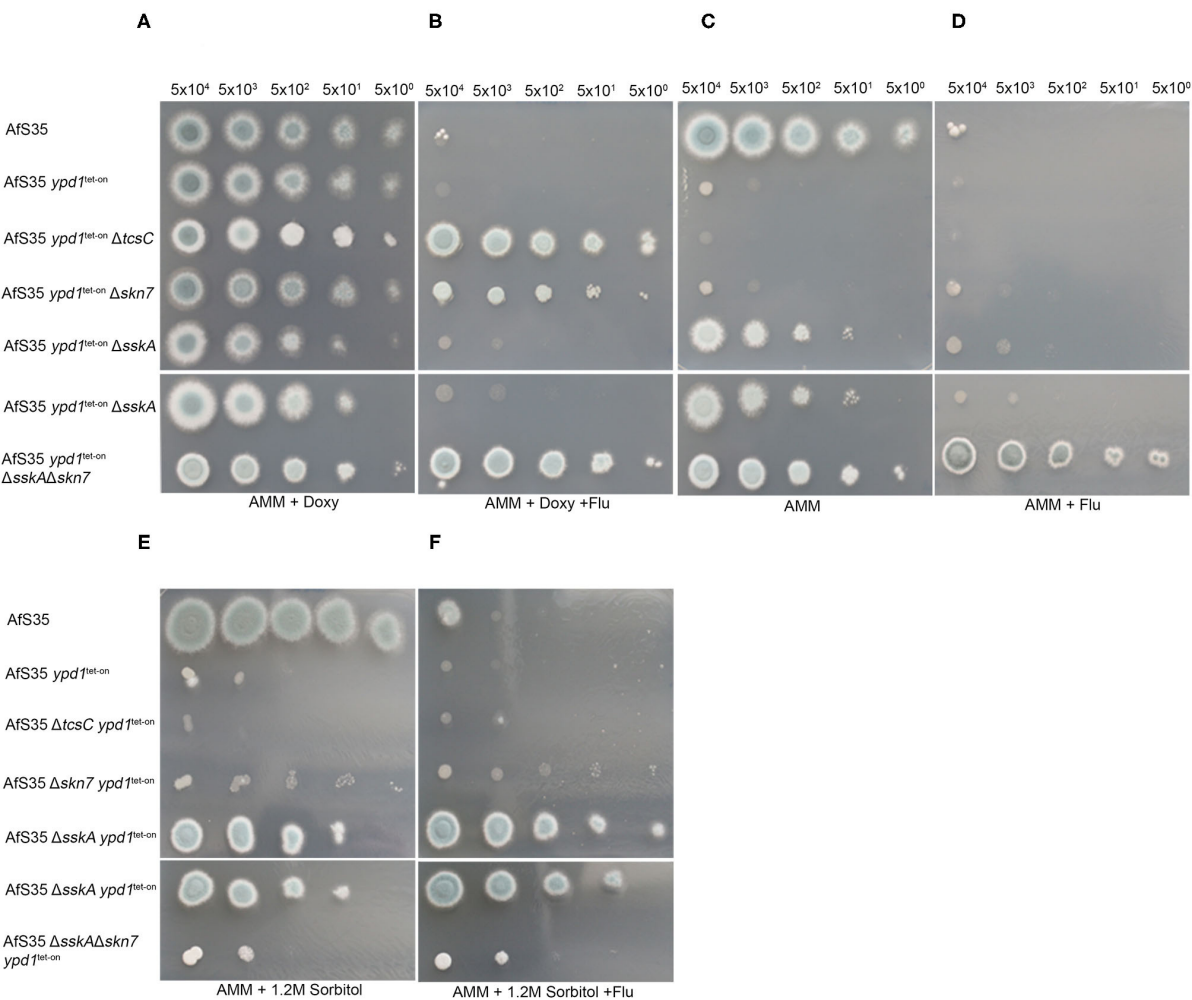
Characterization of *ypd*1^tet−on^ variants of different HOG mutants. We performed drop dilution assays with the indicated numbers of conidia per spot on AMM plates supplemented with doxy (5 μg/ml), fludioxonil (1 μg/ml) and sorbitol (1.2 M) as indicated. All strains grew well on AMM + doxy **(A)**. Only strains lacking either *ssk*A or *skn7* A were able to grow in the absence of doxy **(C)**. **(B)** Shows that only the *ypd*1^tet−on^ variants of Δ*tcs*C, Δ*skn*7 and Δ*ssk*AΔ*skn*7 are resistant to fludioxonil, when grown in the presence of doxy. Under non-inducing conditions, the Δ*ssk*A *ypd*1^tet−on^ is sensitive to fludioxonil, whereas the Δ*ssk*AΔ*skn*7 *ypd*1^tet−on^ is resistant (compare **C** and **D)**. The sensitivity of the different strains to 1.2 M sorbitol on plates without doxy is shown in **(E)**. **(F)** Demonstrates that the antifungal activity of fludioxonil on the Δ*ssk*A *ypd*1^tet−on^ strain can be overcome by osmoprotection.

The importance of Ypd1 for the viability of *A. fumigatus* suggests that a permanent phosphotransfer to the RRs is crucial to avoid a fatal activation of the HOG-pathway. To investigate this further, we generated *ypd*1^tet−on^ mutants in strains lacking either *tcs*C, *ssk*A or *skn*7. In the presence of doxy, these double mutants showed a normal growth; the resulting colonies sporulated well, only the Δ*tcs*C *ypd*1^tet−on^ stood out due to its characteristic white rim (Figure 5A, upper panel). As expected, we found that the absence of doxy strongly impaired the growth of the AfS35 *ypd*1^tet−on^ strain and similar growth defects were detectable for Δ*skn*7 *ypd*1^tet−on^ and Δ*tcs*C *ypd*1^tet−on^ (Figure 5C; upper panel). This indicates that neither TcsC nor Skn7 are essentially required for the deleterious effect caused by a reduced *ypd*1 expression. Colonies of the Δ*tcs*C *ypd*1^tet−on^ strain obtained under non-inducing conditions were even more impaired in growth than those of the other two mutants and individual hyphae of this strain appeared to be also more distorted (Figure 5C, upper panel and Supplementary Figure 6).

The Δ*ssk*A *ypd*1^tet−on^ mutant grew well on AMM plates without doxy and the same applied to the Δ*ssk*AΔ*skn*7 *ypd*1^tet−on^ triple mutant (Figure 5A). This indicates that SskA is critical for the deleterious phenotype caused by a reduced *ypd*1 expression level. On the one hand, this fits well to a similar finding previously reported for *S. cerevisae* (Posas et al., 1996) on the other hand, this result contrasts to our recent finding that the antifungal activity of fludioxonil in *A. fumigatus* is primarily due to Skn7 and depends only to a lesser extent on SskA (Schruefer et al., 2021) (Figure 5B).

Remarkably, the Δ*ssk*A *ypd*1^tet−on^ mutant grown under non-inducing conditions was clearly sensitive to fludioxonil (Figure 5D, upper panel). As in the wild type, the lethal activity of fludioxonil depends on Skn7, which is evidenced by the fludioxonil resistance of the Δ*ssk*AΔ*skn*7 *ypd*1^tet−on^ triple mutant (Figure 5D, lower panel). The presence of 1.2 M sorbitol impaired the growth of all strains except AfS35 and the Δ*ssk*AΔ*skn*7 *ypd*1^tet−on^ strain (Figure 5E). In the presence of fludioxonil, only the *ssk*A deficient strains were osmoprotected by sorbitol (Figure 5F). We have recently shown that fludioxonil causes only a limited increase of the internal glycerol concentration, if the SskA/SakA axis is inactivated (Schruefer et al., 2021) and this moderate Skn7-dependent response can be compensated by osmoprotection.

**Figure 6:**
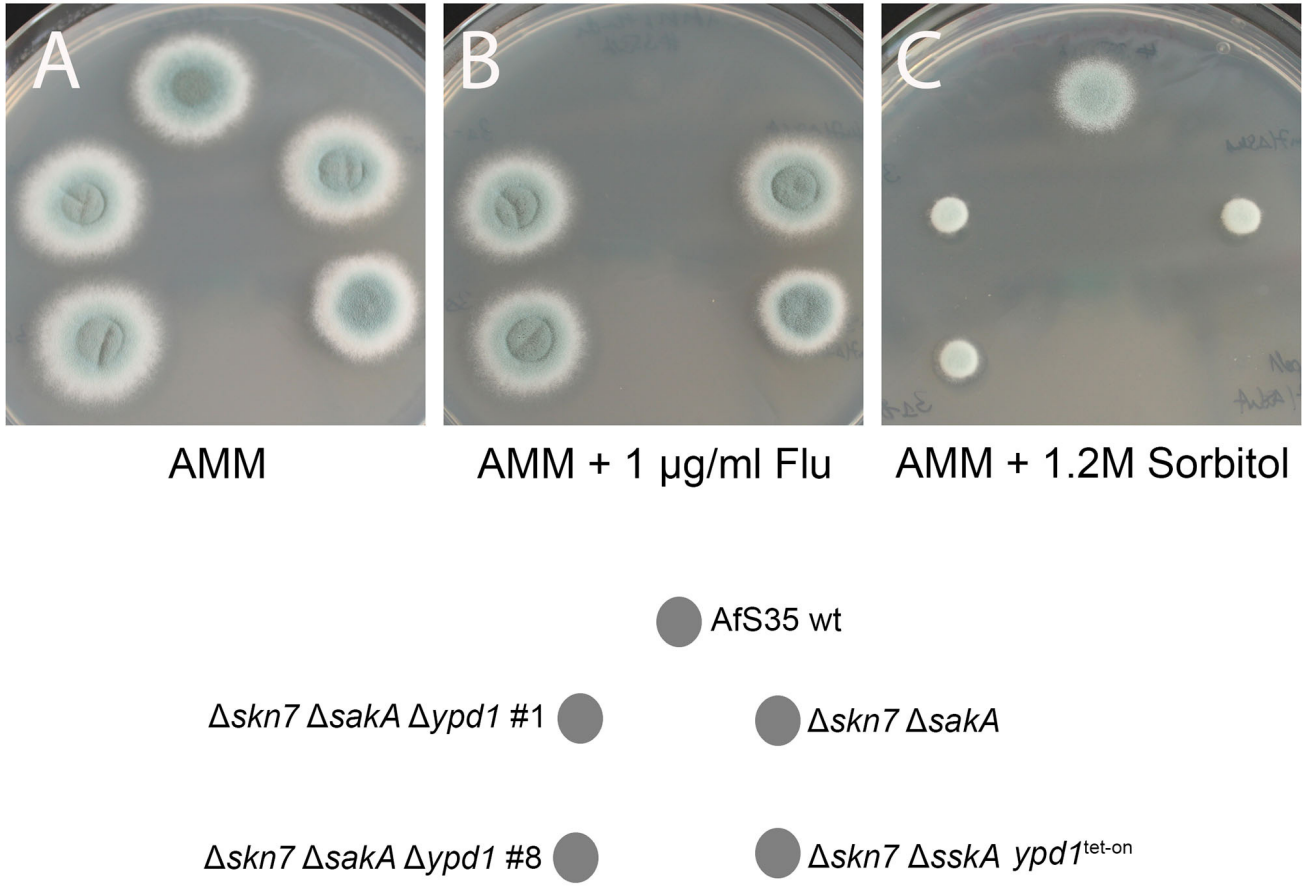
Two independent clones of the Δ*ypd*1Δ*sak*AΔ*skn*7 triple mutant (#1 and #8) show a normal growth on AMM plates **(A)**. As its parental strain Δ*sak*AΔ*skn*7, the triple mutants are resistant to fludioxonil **(B)** and sensitive to hyperosmotic stress (1.2 M sorbitol; **C**). The Δ*skn*7Δ*ssk*A *ypd*1^tet−on^ strain was also analyzed for comparison.

The fludioxonil sensitivity of the Δ*ssk*A *ypd*1^tet−on^ mutant suggests that the impact of fludioxonil is either independent of Ypd1 or requires only low amounts of Ypd1. To address this point, we tried to delete *ypd*1 in a Δ*ssk*A background. In four independent experiments we obtained no *ypd*1 deletion (data not shown). In a similar attempt using the Δ*sak*AΔ*skn*7 mutant, we obtained several Δ*ypd*1 clones already in the first experiment. The resulting triple mutant strains (clones #1 and #8) grew normal on AMM plates, were sensitive to hyperosmotic stress and resistant to fludioxonil and thereby resembled their parental Δ*sak*AΔ*skn*7 strain (Figures 6A–C).

In conclusion, our data indicate that a deletion of *ypd*1 is lethal as long as an intact *skn*7 gene is present. We assume that, under non-inducing conditions, the Δ*ssk*A *ypd*1^tet−on^ mutant contains a reduced amount of Ypd1 proteins that are sufficient and necessary to keep Skn7 in an inactive state. The sensitivity of Δ*ssk*A *ypd*1^tet−on^ to fludioxonil suggests that the antifungal activity of the agent is mediated by a TcsC-mediated inactivation of Ypd1 that results in a lethal activation of Skn7.

## Discussion

The HOG pathway enables fungi to adapt to a hyperosmotic environment and to other stress conditions. This pathway and in particular its group III HHK is, moreover, an attractive target for novel therapeutic agents, for which the bacterial compound pyrrolnitrin and its derivative fludioxonil are lead substances. *A. fumigatus* is a major fungal pathogen and its group III HHK TcsC is both, the main sensor protein of the HOG pathway and a key factor in the antifungal activity of fludioxonil and pyrrolnitrin (McCormick et al., 2012; Hagiwara et al., 2013).

The HOG pathway was described in detail in the model organism *S. cerevisiae*. A major difference between baker's yeast and *A. fumigatus* exists at the level of the HOG sensor kinase: *S. cerevisiae* Sln1p is a group VI and TcsC a group III HHK. Loss of Sln1p is lethal (Maeda et al., 1994), whereas a Δ*tcs*C mutant can grow normally under ambient conditions (McCormick et al., 2012; Hagiwara et al., 2013). Another distinctive feature is that *S. cerevisiae* lacks group III HHKs. TcsB, the *A. fumigatus* ortholog of Sln1p, shares 38.76% identical residues and all functional domains with Sln1p: an N-terminal section containing two transmembrane regions, a histidine kinase domain and a receiver domain (Ota and Varshavsky, 1993). In contrast to Sln1p, TcsB is not essential for *A. fumigatus* and a *tcs*B deletion mutant had no discernible phenotype apart from a slightly enhanced sensitivity to SDS (Du et al., 2006).

The general organization of the HOG pathway seems to be well-conserved in the fungal kingdom. In *A. fumigatus*, the initial multistep phosphorelay comprises of TcsC, the HPt protein Ypd1 and the two RRs SskA and Skn7. The basic concept of the phosphorelay is that the HPt protein phosphorylates and thereby inactivates the two RRs under ambient conditions, although *S. cerevisiae* Skn7p was shown to be activated, rather than inactivated by phosphorylation (Ketela et al., 1998; Li et al., 1998).

In this study, we have initially analyzed the localization of Ypd1, SskA and Skn7. SskA, which controls the activity of a downstream MAPK cascade, was found to reside in the cytosol, whereas Skn7 was highly enriched in the nuclei. This complementary pattern was not affected by fludioxonil and resembles that previously reported for *S. cerevisiae* Ssk1p and Skn7p in the presence and absence of hyperosmotic stress (Lu et al., 2003). GFP-Ypd1 was detected both, in the nucleus and the cytoplasm and thereby resembles *S. cerevisiae* Ypd1p (Lu et al., 2003). Treatment with fludioxonil did not result in a dramatic change of this nucleocytoplasmic pattern. The presence of its two target molecules in the cytosol and the nucleus requires Ypd1 to shuttle between both cellular compartments. After bleaching of the cytosolic pool of GFP-Ypd1, we indeed found that the resulting nuclear enrichment of the GFP signals declined over time and disappeared after 10–15 min. This indicates that GFP-Ypd1 is able to shuttle between the nucleus and the cytoplasm.

Evidence from the related fungus *A. nidulans* suggested that Ypd1 could be an essential protein. We took this into account and generated *ypd*1^tet−on^ mutants in which the native *ypd*1 promotor was replaced by a tet-on construct. Under non-inducing conditions (in the absence of doxycyclin), the wild type (AfS35) *ypd*1^tet−on^ strain was hardly able to form colonies and its hyphae were swollen and often showed signs of cellular lysis. This suggested that Ypd1 is an essential protein in *A. fumigatus*.

To characterize the role of other proteins of the multistep phosphorelay, we established *ypd*1^tet−on^ derivatives of several deletion mutants. Strains with a *tcs*C or *sk*n7 deletion showed a similar phenotype as AfS35 *ypd*1^tet−on^ demonstrating that neither TcsC nor Skn7 are essentially required for the deleterious process that results from a reduced *ypd*1 expression level. In *C. neoformans, ypd*1 is an essential gene, but a deletion strain can be obtained in a strain lacking *hog*1 to prevent a lethal activation of the HOG pathway (Lee et al., 2011). In this study, we found that an *ypd*1^tet−on^ mutant is viable in a strain that also lacks *ssk*A. This suggests that the low levels of Ypd1, which are present in an *ypd*1^tet−on^ strain under non-inducing conditions, can control Skn7 but not SskA. The phenotype associated with a depletion of Ypd1 comprises a dramatic swelling of the fungal cells, an enhanced septation, an elevated number of nuclei per compartment as well as an increased chitin content in the cell wall, all features previously described for fludioxonil-treated *A. fumigatus* wild type hyphae (McCormick et al., 2012; Wiedemann et al., 2016). Very recently, Yoshimi et al. (2021) also reported that a downregulation of *ypd*A expression results in growth inhibition and aberrant hyphal morphology in *A. nidulans*.

We have recently shown that the antifungal activity of fludioxonil is largely dependent on Skn7 and only to a minor extend on the SskA - SakA axis (Schruefer et al., 2021). The Δ*ssk*A *ypd*1^tet−on^ double and the Δ*ssk*AΔ*skn*7 *ypd*1^tet−on^ triple mutant both grow well without doxy, but only the former is sensitive to fludioxonil, which confirms the important role of Skn7 in this context. The fact that this lethal effect can be overcome by osmoprotection may be explained by the moderate increase of the internal glycerol concentration previously found in a Δ*ssk*A strain (Schruefer et al., 2021).

Similar to its counterpart in *S. cerevisiae, A. fumigatus* Ypd1 is apparently required to control the activity of SskA and Skn7 and the fact that the conserved His residue at position 89 is important for this biological activity suggests that Ypd1 transfers phosphoryl groups to the RRs and most likely to conserved aspartate residues in their receiver domains. For this activity, Ypd1 itself has to be constantly phosphorylated under ambient conditions. In *S. cerevisiae*, this is done by Sln1p, which consequently is an essential protein. In *A. fumigatus*, TcsC is dispensable under ambient conditions and has therefore a clearly different function than Sln1p. Since individual knock-out strains in all 13 HHK genes of *A. fumigatus* are viable (Chapeland-Leclerc et al., 2015), it appears likely that Ypd1 receives phosphoryl groups from more than one HHK; whether TcsC is involved remains an open question.

The data obtained with the *ypd*1^tet−on^ construct already suggested that Ypd1 is essential in *A. fumigatus*. The viability of the Δ*ssk*A *ypd*1^tet−on^ mutant either suggests that an *ypd*1 deletion is tolerated in this genetic background or that the low amounts of Ypd1 in this mutant are sufficient to enable its growth. To address this point, we tried to establish an *ypd*1 deletion of the Δ*ssk*A strain. This attempt failed in four independent experiments, which strongly suggests that, in a Δ*ssk*A background, a certain amount of Ypd1 is required to control the Skn7 activity. This concept is corroborated be the fact that we easily obtained *ypd*1 deletion mutants in a Δ*sak*AΔ*skn*7 background.

**Figure 7:**
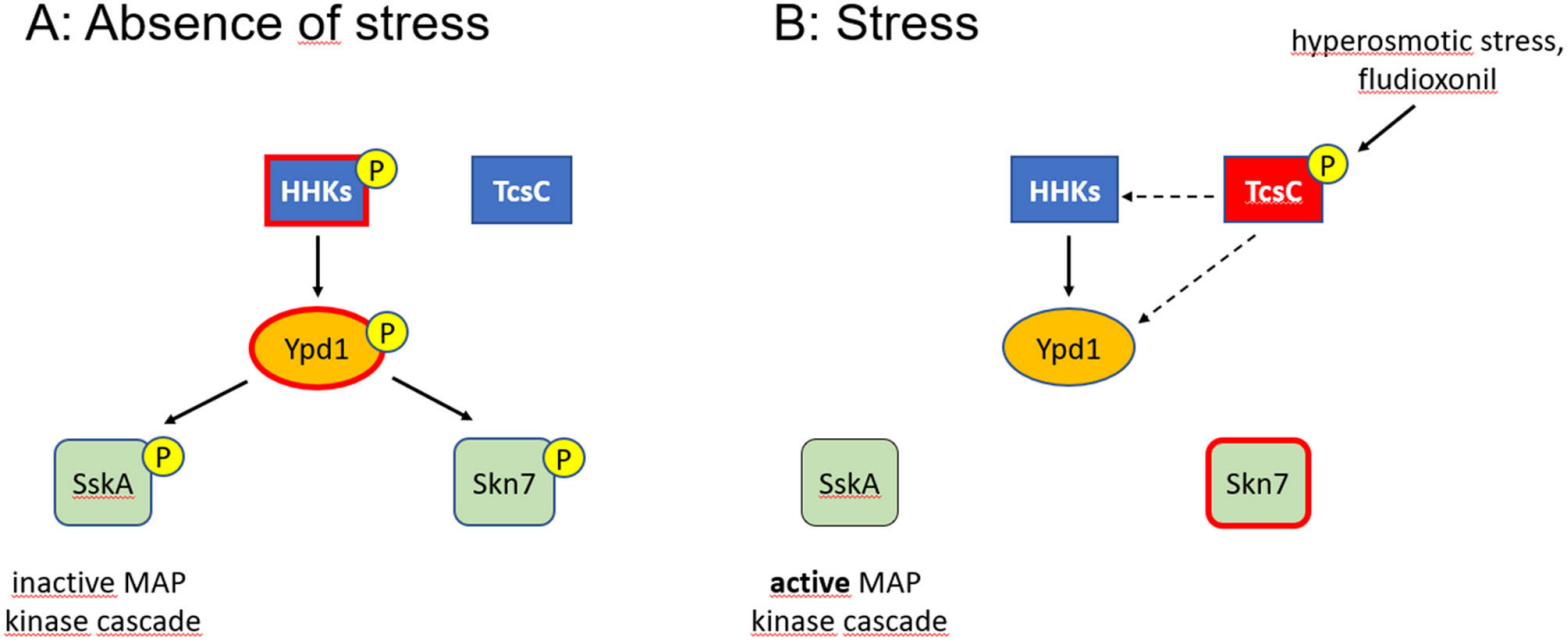
Model of the multistep phosphorelay of *A. fumigatus*. Phosphorylated proteins are indicated by a (P), activated proteins by a red edge. In the absence of stress, Ypd1 is phosphorylated (most likely at residue His_89_) and inactivates the two RRs SskA and Skn7 by phosphorylation **(A)**. The similar phenotypes observed for fludioxonil-treated and Ypd1-depleted hyphae suggest that activated TcsC inactivates Ypd1 either directly or through interactions with those HHKs that phosphorylate Ypd1 (dashed arrows) **(B)**. Whether a phosphorylation by Ypd1 results in an inactivation of Skn7, as indicated in **(A)**, is still an open issue.

While the core HOG pathway is well conserved in the fungal kingdom, it is still unclear which HHK(s) control Ypd1 (Day and Quinn, 2019). An activation of the HOG pathway, either by fludioxonil or environmental stress, requires TcsC. The fact that fludioxonil treatment and an Ypd1 depletion result in similar phenotypes strongly suggests that TcsC activation entails an inactivation of Ypd1 and consequently a dephosphorylation of the two RRs. This implies that the RRs are active in their de-phosphorylated form as indicated in the model depicted in Figure 7. This concept is well established for Ssk1/SskA, but not for Skn7. Data from *S. cerevisiae* rather indicate a positive regulation of the Skn7 activity by a phosphorylation of the conserved Asp residue at position 427 (Ketela et al., 1998; Li et al., 1998). Further experiments are clearly required to address this point properly.

Our data show that the His_89_ residue in Ypd1 is essentially required to keep the HOG pathway in an inactive state. If TcsC is indeed a histidine kinase, its activation must result in a different phosphorylation, e.g., of an alternative His residue in Ypd1. This could result in a conformational change that prevents a phosphorylation at His_89_ or it may lead to a different specificity of Ypd1 resulting in a phosphorylation of Skn7 instead of SskA. The Ypd1 of *A. fumigatus* harbors three His residues at positions 89, 106 and 112. His_106_ is not well conserved in the Ypd1 proteins of different Aspergilli, leaving only His_112_ as a potential alternative target site. Another possible way to inactivate Ypd1 is de-phosphorylation. Recent data published for the group III HHK Drk1 of *Blastomyces dermatitidis* indicate that this protein possesses a histidine kinase activity under ambient conditions, but can adopt a phosphatase activity under fludioxonil treatment (Lawry et al., 2017).

A role of TcsC in the activation of the HOG pathway is evident, but its target molecules and its precise mode of action remain elusive. Whether TcsC has a functional role under steady state conditions is also unclear, but the distinct morphology of the Δ*tcs*C colonies (McCormick et al., 2012) suggests this. Skn7 is a crucial player in the antifungal activities trigged by fludioxonil (Schruefer et al., 2021). TcsC modulates the activity of Skn7 most likely *via* Ypd1. The similar phenotypes of fludioxonil-treated and Ypd1-depleted hyphae suggest that both processes are characterized by an inactivation of Ypd1. A switch between a kinase and a phosphatase activity as described for *B. dermatitis* Drk1 could explain how TcsC inactivates Ypd1. In the absence of stress, Ypd1, is most likely controlled by two or multiple HHKs (Figure 7); the identification of these HHKs and the analysis of their individual contribution will be an important task for future studies. Hence, even after more than 25 years of research on the HOG pathway (Brewster and Gustin, 2014), many questions are still awaiting an answer.

## Data Availability Statement

The original contributions presented in the study are included in the article/Supplementary Material, further inquiries can be directed to the corresponding author/s.

## Author Contributions

SS performed most experiments. AS performed the localization experiments with GFP-Ypd1. CK performed the localization experiments with GFP-Skn7 and SskA-GFP. LS constructed the ypd1 tet-on construct. SS and FE designed the experiments and wrote the manuscript. All authors contributed to the article and approved the submitted version.

## Funding

This work was funded by a grant of the Deutsche Forschungsgemeinschaft (EB-184-1) to FE.

## Conflict of Interest

The authors declare that the research was conducted in the absence of any commercial or financial relationships that could be construed as a potential conflict of interest.

## Publisher's Note

All claims expressed in this article are solely those of the authors and do not necessarily represent those of their affiliated organizations, or those of the publisher, the editors and the reviewers. Any product that may be evaluated in this article, or claim that may be made by its manufacturer, is not guaranteed or endorsed by the publisher.

## Supplementary Material

Supplementary Table 1 Strains used in this study.

Supplementary Table 2 Oligonucleotides used in this study. Letters in lower case indicate mutated bases. Bold letters indicate SfiI restriction sites.

Supplementary Figure 1 Localization of GFP-Ypd1 in hyphae also stained with DAPI. **(A,B)** show a control hypha, whereas **(C,D)** were taken after 4 h incubation with 2 μg/ml fludioxonil. All images are single optical planes taken with a confocal microscope. The position of individual nuclei is indicated by arrows.

Supplementary Figure 2 Localization of GFP-Skn7 **(A–B****″****)** and SskA-GFP **(C–D****″****)** in normal hyphae **(A–A****″****, C–C****″****)** and hyphae treated for 4 h with 1 μg/ml fludioxonil **(B–B****″****, D–D****″****)**. SskA-GFP was cytosolic, whereas GFP-Skn7 showed a clearly nuclear localization. Both patterns were not affected by fludioxonil (compare **A****′****–B****′** and **C****′****–D****′**). The images show single optical planes taken from confocal stacks.

Supplementary Figure 3 Growth and fludioxonil sensitivity of the AfS35 *ypd*1^tet−on^ strain in the presence of different doxy concentrations. Both panels show 6 well tissue culture plates that contained AMM agar supplemented with the indicated concentrations of doxy (in μg/ml). The agar in the lower plate contained 1 μg/ml of fludioxonil. 5 × 10^4^ conidia were applied per spot and the plates were incubated at 37°C for 48 h. The few white colonies that appeared after growth on fludioxonil-containing agar supplemented with 0.5 to 5 μg/ml doxy are most likely spontaneous mutants.

Supplementary Figure 4 Alignment of the protein sequences of *S. cerevisiae* Ypd1p (YDL235C), *A. fumigatus* Ypd1 (Afu4g10280) and *A. nidulans* YpdA (AN2005). The conserved histidine residue in the HPT domain is indicated in red. The HPT domain itself is underlined.

Supplementary Figure 5 qPCR analysis of *ypd*1 expression in AfS35 *ypd*1 ^tet−on^ and its derivatives expressing either wild type *ypd*1 or *ypd*1 harboring the H89G point mutation. All strains were grown in AMM supplemented with 0.5 μg/ml doxy.

Supplementary Figure 6 **(A)** shows colonies of the indicated mutants that were grown for 48 h on AMM plates without doxy. The microscopic images show bright field images **(B,D,F)** and CFW staining **(C,E,G)** of hyphae from the edge of these colonies. The arrows in **(F)** indicate hyphal tips showing an unusual branching pattern.
