# Supplementary material for: Ypd1 Is an Essential Protein of the Major Fungal Pathogen Aspergillus fumigatus and a Key Element in the Phosphorelay That Is Targeted by the Antifungal Drug Fludioxonil
Source: Front Fungal Biol. 2021 Oct 18;2:756990. doi: 10.3389/ffunb.2021.756990 (PMC10512271; doi:10.3389/ffunb.2021.756990)
Supplement: Supplementary Table 1 — Strains used in this study. [file Data_Sheet_1.PDF]

**Strain**	**Source**	**Resistance markers**
AfS35	Krappmann *et al*., 2006	
AfS35 *ypd*1-*gfp*	this study	pyrithiamine
AfS35 *gfp-ssk*A	this study	pyrithiamine
AfS35 *gfp-skn*7	this study	pyrithiamine
Δ*tcs*C	McCormick *et al*., 2012	hygromycin B
Δ*skn*7	Schruefer *et al*., 2021	hygromycin B
Δ*ssk*A	Schruefer *et al*., 2021	hygromycin B
Δ*skn*7Δ*sak*A	Schruefer *et al*., 2021	hygromycin B
AfS35 *ypd*1$^{\text{tet-on}}$	this study	pyrithiamine
AfS35 *ypd*1$^{\text{tet-on}}$ + *rfp-stu*A	this study	pyrithiamine, phleomycin
AfS35 Δ*tcs*C *ypd*1$^{\text{tet-on}}$	this study	hygromycin B, pyrithiamine
AfS35 Δ*skn*7 *ypd*1$^{\text{tet-on}}$	this study	hygromycin B, pyrithiamine
AfS35 Δ*ssk*A *ypd*1$^{\text{tet-on}}$	this study	hygromycin B, pyrithiamine
AfS35 Δ*skn*7Δ*sak*A *ypd*1$^{\text{tet-on}}$	this study	hygromycin B, pyrithiamine
Δ*ssk*AΔ*ypd*1	this study, not viable	hygromycin B, pyrithiamine
Δ*skn*7Δ*sak*AΔ*ypd*1	this study	hygromycin B, pyrithiamine
AfS35 *ypd*1$^{\text{tet-on}}$ + *ypd*1	this study	pyrithiamine, phleomycin
AfS35 *ypd*1$^{\text{tet-on}}$ + *ypd*1$^{\text{H89G}}$	this study	pyrithiamine, phleomycin

Suppl. Table 1: Strains used in this study
