# Supplementary material for: Ypd1 Is an Essential Protein of the Major Fungal Pathogen Aspergillus fumigatus and a Key Element in the Phosphorelay That Is Targeted by the Antifungal Drug Fludioxonil
Source: Front Fungal Biol. 2021 Oct 18;2:756990. doi: 10.3389/ffunb.2021.756990 (PMC10512271; doi:10.3389/ffunb.2021.756990)
Supplement: Supplementary Table 2 — Oligonucleotides used in this study. Letters in lower case indicate mutated bases. Bold letters indicate SfiI restriction sites. [file Data_Sheet_2.PDF]

Designation	Sequence
GFP-ypd1-For	GATGGCTCCTACAACCACT
ypd1-For	ATGGCTCCTACAACCACT
ypd1-Rev	CTATTTAGAGGAGGGCGA
sskA-GFP-For	ATGCCTGACCGCCGCCTG
sskA-GFP-Rev	GCCGTAGCATCCAGAGC
GFP-skn7-For	GGAGGGTGGCCAGACCAC
GFP-skn7-REV	TTAGCCACTTCGAGTAGC
ypd1-up-FOR	AGAACCATACAAGGTGGGAT
ypd1-up-Rev tet-on	**TCCGGTAGATCCGG**CCTCCTCGTGGCGTATGT
ypd1-FOR-SfiI	C**GGCCTGAGTGGCC**ATGGCTCCTACAACCACTAC
ypd1-do-Rev tet-on	CTATGAATCATGGCCCATACA
stuA-in-EcoRV-FOR	ATGTCCTACACCTACACCA
stuA-in-EcoRV-REV	TCATCGACGACGAGGCATG
ypd1 deletion up For	ACAAGGTGGGATACCCCAATC
ypd1 deletion up Rev	CTCCTCCTCGTGGCGTATGTG
ypd1 deletion do For	TGATTGATCAAGGGGGAAGAG
ypd1 deletion do Rev	CTACGCGCGCCCTCAAG
Pyrith cassette For	TTGATTACGGGATCCCATTGG
Pyrith cassette Rev	TTACTCAGCACACTCGCGC
ypd1 H89G For	GAAAGCTCATCGAGGTCC
ypd1 H89G Rev	TTCGCTTGGAggtTTCCTAAAAGGATCGTC
ypd1 qpcr for2	TGAGGAATCTCCCCAACCCAA
ypd1 qpcr rev2	CGTCCATCTCCAAGATCTGGTCA
tubA qpcr for	ACGGCGGAAACACGGAAAAC
tubA qpcr rev	CGAGATCGACCAGAACGGCA

Suppl. Table 2: Oligonucleotides used in this study

Letters in lower case indicate mutated bases. Bold letters indicate SfiI restriction sites.
