# Supplementary figures and images for: Ypd1 Is an Essential Protein of the Major Fungal Pathogen Aspergillus fumigatus and a Key Element in the Phosphorelay That Is Targeted by the Antifungal Drug Fludioxonil
Source: Front Fungal Biol. 2021 Oct 18;2:756990. doi: 10.3389/ffunb.2021.756990 (PMC10512271; doi:10.3389/ffunb.2021.756990)

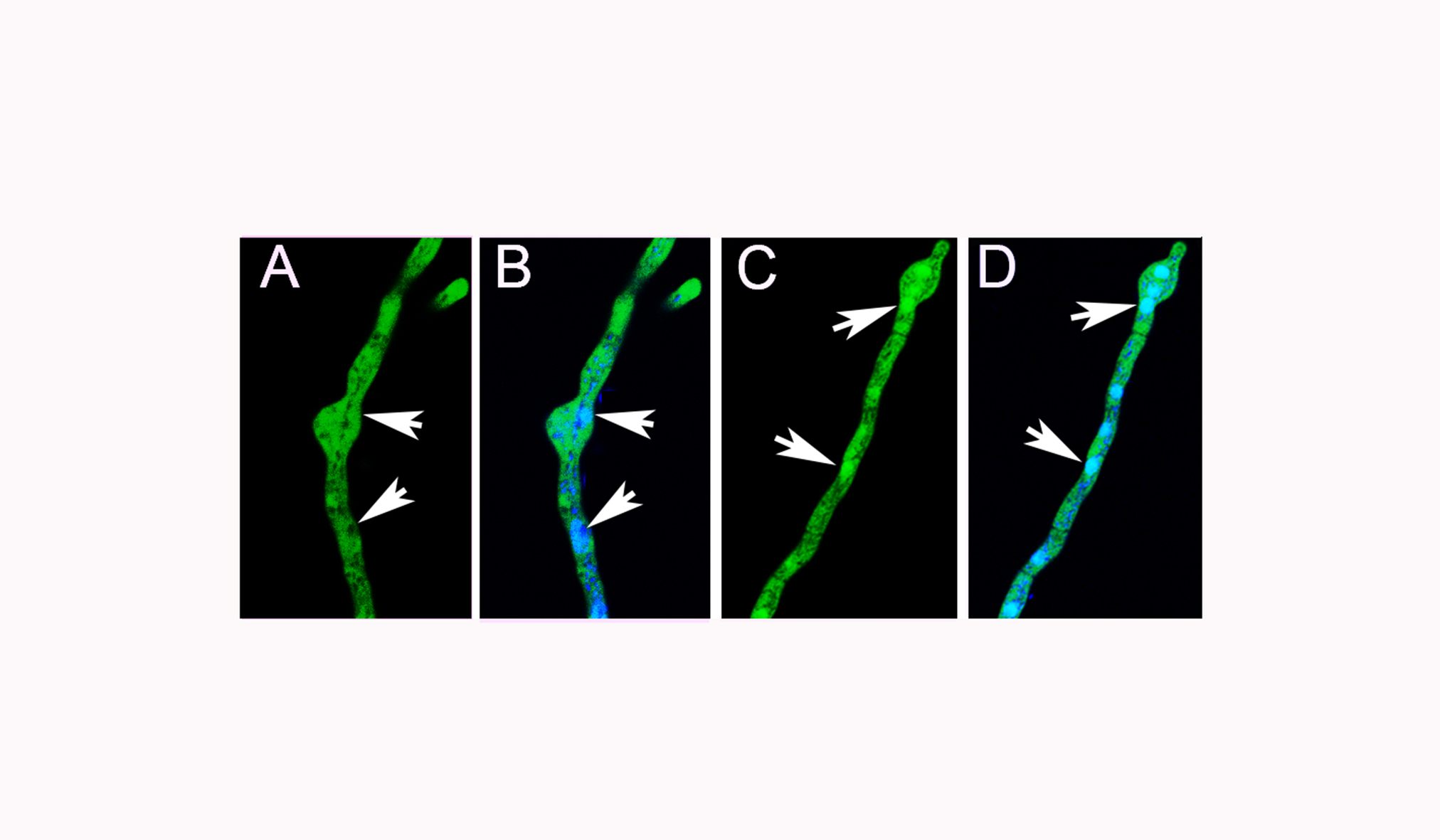

Supplement: Supplementary Figure 1 — Localization of GFP-Ypd1 in hyphae also stained with DAPI. (A,B) show a control hypha, whereas (C,D) were taken after 4 h incubation with 2 μg/ml fludioxonil. All images are single optical planes taken with a confocal microscope. The position of individual nuclei is indicated by arrows. [file Image_1.JPEG]

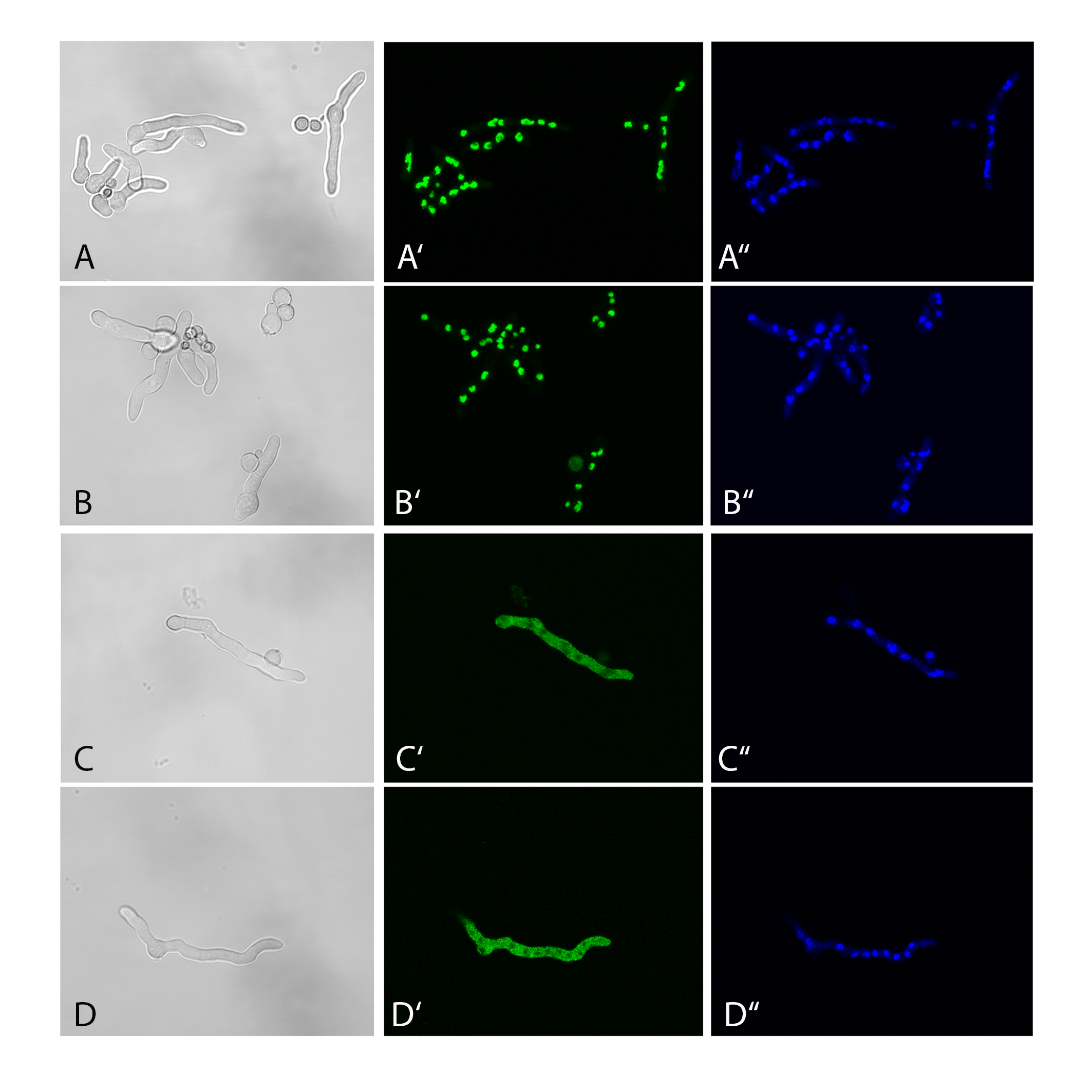

Supplement: Supplementary Figure 2 — Localization of GFP-Skn7 (A–B″) and SskA-GFP (C–D″) in normal hyphae (A–A″, C–C″) and hyphae treated for 4 h with 1 μg/ml fludioxonil (B–B″, D–D″). SskA-GFP was cytosolic, whereas GFP-Skn7 showed a clearly nuclear localization. Both patterns were not affected by fludioxonil (compare A′–B′ and C′–D′). The images show single optical planes taken from confocal stacks. [file Image_2.TIF]

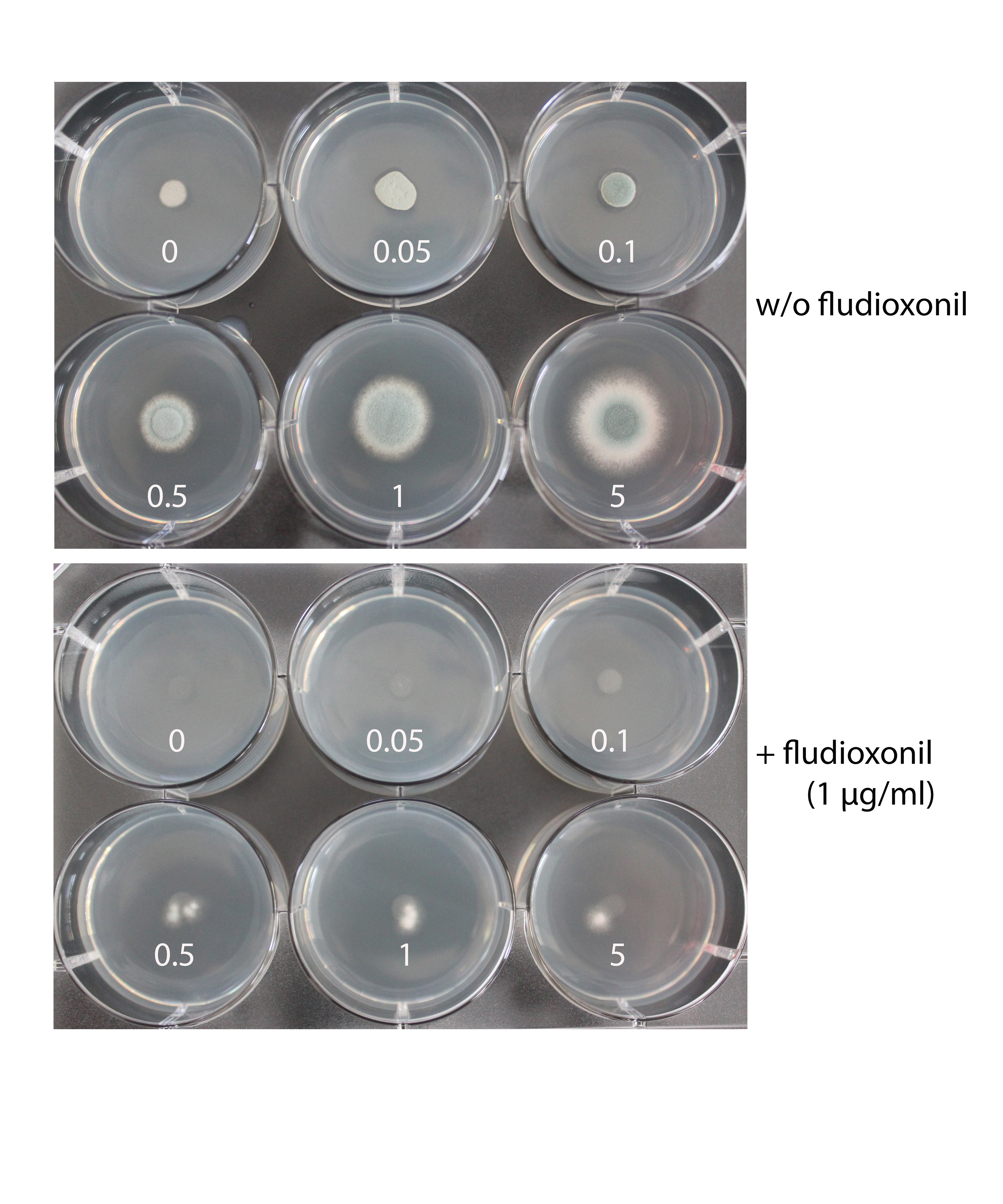

Supplement: Supplementary Figure 3 — Growth and fludioxonil sensitivity of the AfS35 ypd1tet−on strain in the presence of different doxy concentrations. Both panels show 6 well tissue culture plates that contained AMM agar supplemented with the indicated concentrations of doxy (in μg/ml). The agar in the lower plate contained 1 μg/ml of fludioxonil. 5 × 10^4^ conidia were applied per spot and the plates were incubated at 37°C for 48 h. The few white colonies that appeared after growth on fludioxonil-containing agar supplemented with 0.5 to 5 μg/ml doxy are most likely spontaneous mutants. [file Image_3.JPEG]

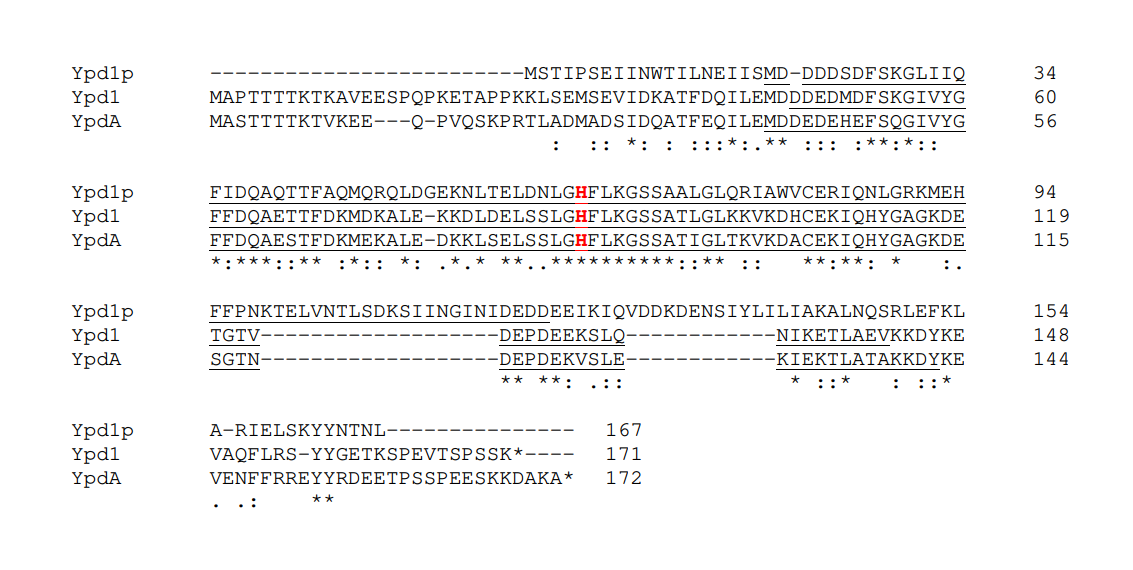

Supplement: Supplementary Figure 4 — Alignment of the protein sequences of S. cerevisiae Ypd1p (YDL235C), A. fumigatus Ypd1 (Afu4g10280) and A. nidulans YpdA (AN2005). The conserved histidine residue in the HPT domain is indicated in red. The HPT domain itself is underlined. [file Image_4.TIF]

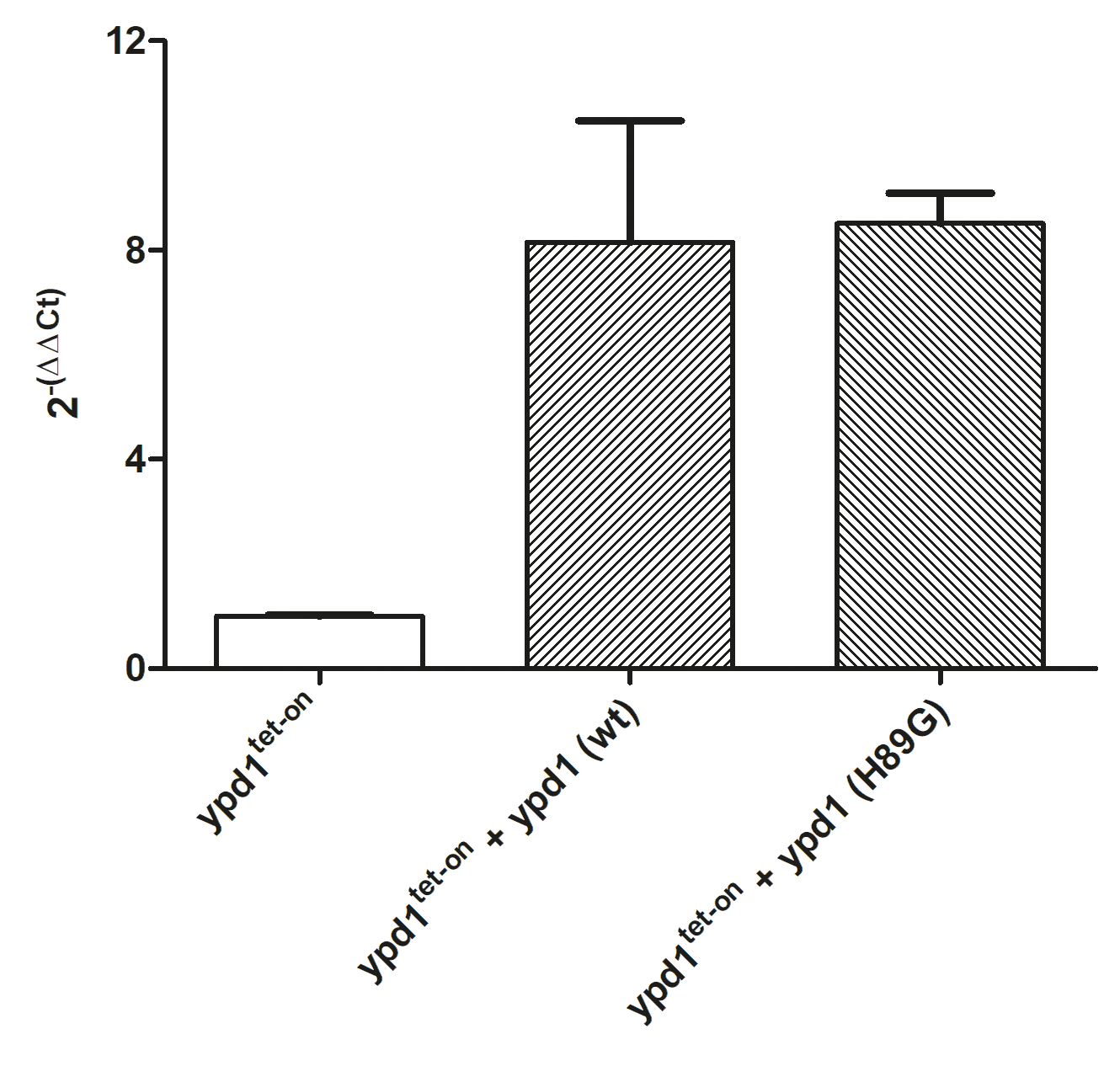

Supplement: Supplementary Figure 5 — qPCR analysis of ypd1 expression in AfS35 ypd1 tet−on and its derivatives expressing either wild type ypd1 or ypd1 harboring the H89G point mutation. All strains were grown in AMM supplemented with 0.5 μg/ml doxy. [file Image_5.TIF]

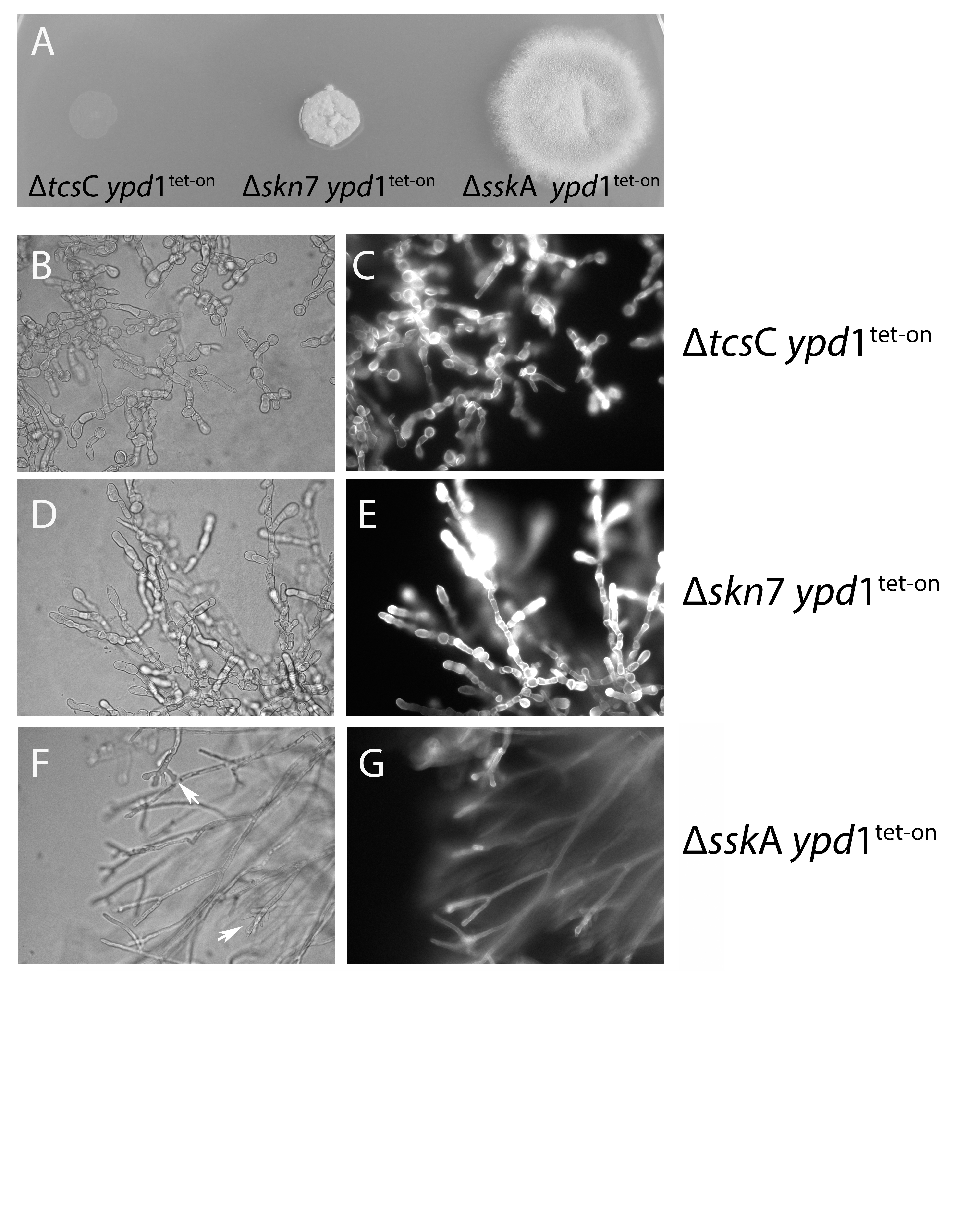

Supplement: Supplementary Figure 6 — (A) shows colonies of the indicated mutants that were grown for 48 h on AMM plates without doxy. The microscopic images show bright field images (B,D,F) and CFW staining (C,E,G) of hyphae from the edge of these colonies. The arrows in (F) indicate hyphal tips showing an unusual branching pattern. [file Image_6.JPEG]
